# Zeaxanthin Dipalmitate in the Treatment of Liver Disease

**DOI:** 10.1155/2019/1475163

**Published:** 2019-08-21

**Authors:** Nisma Lena Bahaji Azami, Mingyu Sun

**Affiliations:** ^1^Key Laboratory of Liver and Kidney Diseases, Institute of Liver Diseases, Shuguang Hospital Affiliated to Shanghai University of Traditional Chinese Medicine, Shanghai 201203, China; ^2^Shanghai University of Traditional Chinese Medicine, Shanghai 201203, China

## Abstract

Goji berry, *Lycium barbarum*, has been widely used in traditional Chinese medicine (TCM), but its properties have not been studied until recently. The fruit is a major source of zeaxanthin dipalmitate (ZD), a xanthophyll carotenoid shown to benefit the liver. Liver disease is one of the most prevalent diseases in the world. Some conditions, such as chronic hepatitis B virus, liver cirrhosis, and hepatocellular carcinoma, remain incurable. Managing them can constitute an economic burden for patients and healthcare systems. Hence, development of more effective pharmacological drugs is warranted. Studies have shown the hepatoprotective, antifibrotic, antioxidant, anti-inflammatory, antiapoptotic, antitumor, and chemopreventive properties of ZD. These findings suggest that ZD-based drugs could hold promise for many liver disorders. In this paper, we reviewed the current literature regarding the therapeutic effects of ZD in the treatment of liver disease.

## 1. Introduction

**Figure 1:**
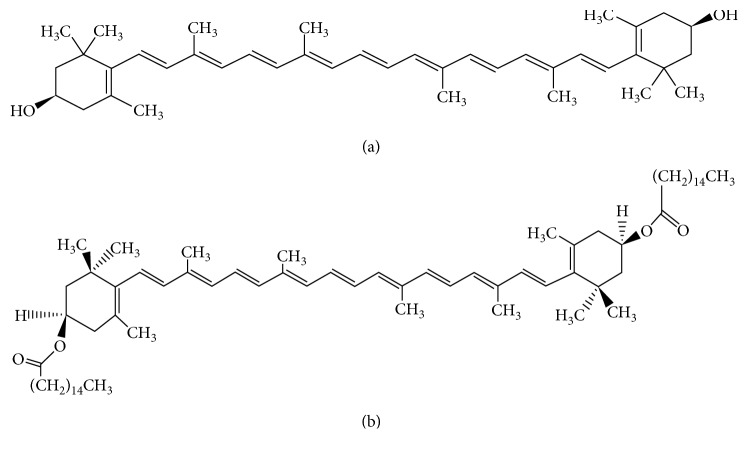
Chemical structures of zeaxanthin and zeaxanthin dipalmitate. (a) Zeaxanthin; (b) zeaxanthin dipalmitate (KNApSAck database).

Goji berry, *Lycium barbarum,* is a popular fruit consumed in China and used in traditional Chinese medicine (TCM). Its description and indications first appeared in *the Divine Husbandman's Classic of Materia Medica* or *Shennong Ben Cao Jing* [1]. In TCM, goji berry treats kidney yin and liver blood deficiencies and their related symptoms such as dry eyes and blurred or decreased vision [1]. Modern research has shown that goji berry protects against oxidative damage [2] and is a rich source of phenolics, linoleic acid, minerals such as potassium and phosphorus [3], flavonoids, as well as polysaccharides [4], such as arabinogalactan [5], and monosaccharides [6]. Goji berry also contains the highest concentration of the antioxidant xanthophyll carotenoid zeaxanthin dipalmitate (ZD) [7]. ZD is a zeaxanthin diester formed from zeaxanthin (ZE) and palmitic acid (Figure 1).

As a vital organ in the human body, the liver plays metabolic, detoxifying, immunological [8], and bile-secreting roles, which are altered under pathogenesis [8–10]. Liver disease is one of the most predominant diseases in the world and is often accompanied by poor prognosis. In China, it affects about 300 million people [11]. So far, there are no pharmaceutical drugs capable of reversing liver fibrosis [12] or curing conditions such as nonalcoholic fatty liver disease (NAFLD), alcoholic liver disease (ALD), hepatocellular carcinoma (HCC), liver cirrhosis (LC), and liver failure (LF). Current prevention and management methods include lifestyle changes, weight loss, and abstinence [13, 14], with liver transplantation being indicated for end-stage liver disease, though patients face the challenge of finding donors and risk postoperative complications [15]. Risk factors, such as obesity, insulin resistance, alcohol consumption, and inadequate resources to prevent viral infections in some areas, contribute to the widespread and progression of liver disease [16–19]. Thus, development of effective drugs is necessary. Recent interest in phytochemicals and TCM has enabled scientists to determine active ingredients in various herbs that could potentially cure liver disease. Besides goji berry-derived ZD, other active compounds have been isolated from TCM herbs, and their effects on liver disease were investigated. These include berberine (Huanglian) [20, 21], emodin (Dahuang) [22], and gastrodin (Tianma) [23, 24]. Although few studies assessing the benefits of ZD cover a wide range of liver conditions. To our best knowledge, this is the first English literature review on the subject. The properties and quantification methods of ZD are first discussed, followed by the potential therapeutic effects of ZD on liver disease. Studies employing ZE instead of ZD are also included.

## 2. Goji Berry

### 2.1. Properties of Goji Berry in TCM and Recent Literature

In TCM, goji berry is used to treat kidney yin deficiency and liver blood deficiency syndromes, which can be discerned in patients through symptom and sign collection, radial pulse palpation, and tongue's shape and fur observation. In the case of kidney yin deficiency, palpating the radial pulse often reveals a thready and rapid pulse. The tongue body is usually dry and red with or without fur. Other clinical signs and symptoms include, but are not limited to, dry mouth and throat, night sweats, dizziness, tinnitus, weak legs and knees, and gynecological disorders such as seminal emission and amenorrhea [25]. Classical herbal formulas to tonify kidney yin such as Zuo Gui Wan (restore the left (kidney) pills) contain goji berry and are currently sold in Chinese pharmacies as OTC drugs. The Zuo Gui Wan formula was first recorded in *The Complete Works of Jing Yue* (*Jing Yue Quan Shu*) by Zhang Jingyue, a famous doctor from the Ming dynasty (1368–1644). Another OTC formula containing goji berry is Yi Guan Jian (ever-effective decoction), which was first recorded in the Qing dynasty and whose current clinical applications include acid regurgitation, gastric ulcer, and chronic hepatitis [26].

Recently, the effect of goji berry and its compounds on different diseases have been elucidated. Evidence showed that goji berry may offer retinal protection and tissue restoration in early stage of type 2 diabetes [27]. Here, ZD lowered endoplasmic reticulum (ER) stress biomarkers and restored the activities of AMP-activated protein kinase (AMPK) and forkhead O transcription factor 3 *α* (FoxO3*α*). In line with these results, a nonpurified phytochemical extract from goji berry was found to have chemopreventive effects and could halt the proliferation of leukemia cells by eliminating oxidative stress and inhibiting the nuclear factor-kappa B (NF-*κ*B) NF-*κ*B and PI3/AKT pathways while activating AMPK [28]. Furthermore, water-soluble *Lycium barbarum* polysaccharides (LBP) induced apoptosis of cervical cancer cells through the collapse of mitochondrial transmembrane potential and the accumulation of intracellular calcium and nitric oxide in cancer cells [29]. In carbon tetrachloride- (CCL4-) induced liver injury, LBP was shown to alleviate fibrosis; reduce hepatic necrosis, serum ALT, and lipid peroxidation; inhibit TLRs/NF-kB signaling pathway; and promote liver regeneration [30, 31]. LBP also displayed cardioprotective activity [32] and were reported to recover testicular function by inhibiting testicular excessive autophagy in an animal model with diabetic testicular dysfunction [33].

### 2.2. Zeaxanthin Dipalmitate Extraction Methods

**Figure 2:**
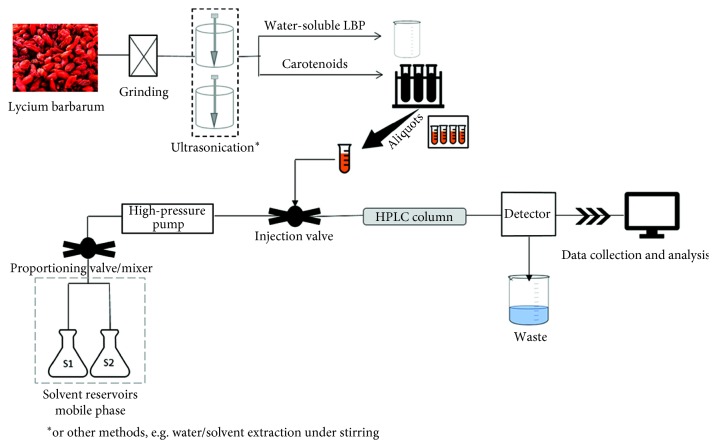
HPLC method for carotenoid quantification.

The ability of goji berry to form chromoplasts enables the fruit to biosynthesize high levels of carotenoids such as ZE and ZD and prevent carotenoid degradation [34]. In fact, adding these carotenoids, including ZD, to cooking oils could prevent their oxidative degradation during storage and improve shelf life, which is a safer alternative to synthetic antioxidants [35]. In ZD, double bond between ZE and palmitic acid can be broken with saponification, in which case free ZE is released [36]. Due to its lipid solubility, the optimal extraction for ZD may be achieved with the use of organic solvents and high-performance liquid chromatography (HPLC) (Figure 2). Acetone is usually employed to isolate ZD because its polarity is closer to the polarity of the latter [37]; however, in the case of an exhaustive extraction of total carotenoid content, other solvents are included [36, 38]. There are many studies in which ZD has been isolated and quantified. We list some of them below (Table 1).

One ZD extraction method, for potentially making dietary supplements, consisted of removing goji berry glycosides, followed by ultrasonic acetone extraction of ZD and column fractioning. The obtained fraction was dissolved in dichloromethane and then precipitated by continuously applying ethanol to recrystallize ZD [37]. A second method consisted of freeze-drying the fruit with nitrogen gas (N2) before grinding it. This was followed by ultrasonication with water to remove polysaccharides and then ultrasonication with hexane/acetone (50 : 50) solvent to extract the carotenoid content. Finally, a mixture of acetone/methanol (55 : 45) was used in the mobile phase of HPLC to isolate ZD. This method showed that ZD constituted more than 85% of the total carotenoid in goji berry [38]. Another method extracting carotenoids using petroleum ether/acetone (1 : 1) followed by acetonitrile/dichloromethane (42 : 58) in the HPLC mobile phase also showed that ZD was the predominant carotenoid (this time 31–56% of total carotenoids) [41]. Similar use of solvents but with the HPLC method consisting of acetonitrile/methylene chloride (60 : 42) in the mobile phase showed that ZD accounted for 77.5% of the total carotenoids [39]. Choosing methanol/ethyl acetate/light petroleum (1 : 1 : 1) to extract ZE and ZE esters from heat-dried goji berries followed by drying the supernatant with anhydrous sodium sulfate and HPLC-DAD analysis with petroleum/acetone (94 : 6) in the mobile phase also revealed that ZD was the major xanthophyll carotenoid in goji berry [40]. Another extraction method involved freezing and storing goji berries at low temperatures before unfreezing and grinding. Extraction of carotenoids was achieved by hydroalcoholic mixture/cyclohexane (1 : 1) and ethyl acetate to prepare the aliquot. The mobile phase consisted of acetone/methanol (80 : 20). This method also validated ZD as the major carotenoid in goji berry (55–81%) [42]. As for carotenoid isolation from goji berry juice, one study showed that hexane/ethanol/acetone (2 : 1 : 1) solvent combination may ensure optimal extraction. The authors of this study reported performing both direct extraction and saponified extraction, followed by UV-visible spectrometry analysis to confirm the profiles of ZD and ZE. Direct extraction was preferred based on optimal carotenoid wavelength absorption. In this case, ZD was also reported as the predominant carotenoid in goji berry juice [36]. It is also worth noting that two geometric isomers of ZD in goji berry have been identified recently: 13Z-zeaxanthin dipalmitate and 9Z-zeaxanthin dipalmitate [43].

### 2.3. ZD Pharmacokinetics

ZD is a polar lipophilic and lipotropic carotenoid obtained through diet and cannot be synthesized by the human body [44]. Although ZD digestion and absorption has not clearly been mapped out, a few studies attempted to elucidate it. *In vitro* experiments showed that lipase and carboxyl ester lipase hydrolyzed ZD into free ZE in the small intestine, where it is absorbed [45, 46]. Furthermore, examination of enterocytes showed no traces of ZD. Rather free ZE and ZE monoesters were present [46]. Another study comparing plasma responses in the human subject after ingestion of ZD and ZE did not detect ZD in the collected blood samples. However, postingestion of ZD correlated with higher plasma ZE compared with consumption of nonesterified ZE. The authors hypothesized that the nonpolar nature of ZD, which allows effective micelle formation before lipase activity, accounts for the results [47]. Correspondingly, supplementing healthy elderly subjects with a milk-based formulation of goji berry for 90 days increased plasma zeaxanthin by 26% and antioxidant activities by 57% [48]. In another study, where volunteers supplemented with goji berry for 28 days, collected blood samples showed fasting plasma ZE increasing by 2.5 folds compared with the baseline [49]. As far as the carotenoids' tissue distribution, a meta-analysis study concluded that ZE along with its isomers lutein (LU) and meso-zeaxanthin (MZ) concentrated in the macula. In addition, the dietary intake of ZE, LU, and MZ increased macular pigment and prevented macular degeneration in healthy individuals and age-related macular degeneration (AMD) patients [50]. Similarly, in a randomized-controlled trial, patients with early AMD consumed goji berries (GB) for 90 days, after which ZE serum levels and macular pigment optical density were significantly higher than the baseline and control group [51]. In animal models, ZE was found in the liver, spleen, and fat and its concentration in these tissues increased after consumption of goji berry [52, 53]. From these studies, we gathered that the uptake of ZD increased the bioavailability of ZE in the blood, macula, spleen, liver, and adipose tissue.

## 3. Zeaxanthin Dipalmitate and Liver Disease

### 3.1. Fibrosis and Biomarkers in Liver Disease

Fibrogenesis or tissue scarring is common in chronic liver disease and may evolve into cirrhosis. As fibrous tissues form in response to injury, the histology and hemodynamics [54] of the liver are altered, impairing its function. During fibrogenesis, Kupffer cells respond to chemokines such as CXCL6 by secreting transforming growth factor-*β*1 (TGF‐*β*1) through the SMAD2/BRD4/C‐MYC/EZH2 pathway [55]. TGF‐*β*1 activates hepatic stellate cells (HSC), which turn into myofibroblasts and shift from storing vitamin A in normal conditions to producing extracellular matrix proteins. In addition, HSC release chemokines and increase the number of inflammatory receptors [56].

So far, there are no FDA-approved drugs for hepatic fibrosis. Nonetheless, studies showed that ZD may ameliorate and inhibit tissue scarring. In fact, histological findings in one animal study showed that oral ZD (25 mg/kg) inhibited secondary fibrosis and decreased the collagen content (including 4-hydroxyproline) in the liver. ZD also restored glutathione S-transferase (GST) activity, which was shown to bind and neutralize bilirubin [57]. In another animal study employing ZE, histopathological assessment indicated that ZE (25 mg/kg) inhibited liver fibrosis [58]. Moreover, treatment with ZD improved hepatic histology and reduced fibrosis in the AFLD model (25 mg/kg) and in the superimposed nonobese nonalcoholic steatohepatitis (NASH) and HBV animal model (2 mg/kg) [59, 60]. Although ZD was shown to attenuate liver fibrosis, the underlying mechanisms are still unclear. Overall, studies have been limited to histological observations. Recent discovery of the role of synectin in fibrogenesis and how this scaffold protein regulates HSC's platelet-derived growth factor receptors (PDGFR), PDGFR-*α*, and PDGFR-*β* may further clarify the mechanisms of fibrosis [61]. Synectin could also be used in future ZD studies to investigate how ZD inhibits fibrosis at a molecular level.

Screening for most hepatic diseases involves measuring the level of serum liver enzymes, which serve as biomarkers of liver injury. Abnormal laboratory results show elevated liver enzymes alanine aminotransferase (ATL) (also called glutamic pyruvic transaminase) and aspartate aminotransferase (AST). In case of hepatobiliary or bone disorders, abnormal levels of alkaline phosphatase (ALP) are also detected [62]. On the contrary, albumin, the most abundant protein in the plasma, decreases in most hepatic diseases, notably in ALD where ethanol inhibits albumin synthesis [63]. In healthy individual, ALT normal range is not standardized and varies from one clinical laboratory to another depending on the local population [64]. The AST : ALT ratio is below 2; otherwise, a ratio exceeding 2 is usually associated with alcoholic hepatitis [65]. Administration of ZD after bile duct ligation induced fibrosis and improved serum ALT, AST, and ALP levels in a dose-dependent manner [57]. ZD also ameliorated serum ALT in the AFLD animal model [59] and serum ALT, AST, ALP, and ALB levels in the superimposed NASH and chronic HBV model [60]. Moreover, concurrent exposure of primary hepatocytes to CCL4 and ZD showed that ZD inhibited the release of ALT and sorbitol dehydrogenase (SDH) [66]. Inhibition of SDH was shown to induce the antiaging and antiapoptotic enzyme SIRT1 and protect the liver against ischemia-reperfusion (I/R) injury and tissue necrosis [67].

Finally, elevated serum levels of total cholesterol and low-density lipoproteins (LDL) are often detected in AFLD and NASH patients. Administration of ZD regulated the imbalanced levels of total cholesterol (TC), triglyceride (TG), high-density lipoprotein (HDL), and low-density lipoprotein (LDL) in NASH and AFLD models [59, 60]. In addition, ZD restored the expression of lipogenic sterol-regulator element binding protein-1c (SREBP-1c) and decreased lipolytic adipose triglyceride lipase ATGL [60].

### 3.2. Nonalcoholic Fatty Liver Disease

The prevalence of nonalcoholic fatty liver disease (NAFLD) has rapidly risen over the past years making the disease one of the leading causes of liver transplant after hepatitis C virus in the United States [68]. The disease is also associated with a high lifetime economic burden for patients [69]. NAFLD features hepatocellular fatty change or steatosis, which can be identified with imaging or biopsy. It can be further divided into nonalcoholic fatty liver (NAFL) and nonalcoholic steatohepatitis (NASH). NAFL tends to be benign and nonprogressive and is characterized by the presence of simple steatosis, or fat infiltration in the hepatocytes, without hepatocyte injury. On the contrary, NASH may evolve into cirrhosis and is characterized by the presence of steatosis and inflammation with hepatocyte injury (such as ballooning) with or without fibrosis [70]. NAFLD risk factors include visceral obesity, excessive BMI, type 2 diabetes mellitus, high HDL levels, and dyslipidemia [71]. Proinflammatory factors such as cytokines and oxidative stress may contribute to the progression of NAFL into NASH. As there is no cure for NAFLD, only lifestyle changes or bariatric surgery to reduce visceral obesity can inhibit inflammation and reverse steatosis [72]. The pathogenesis of NAFLD remains unclear; however, different hypotheses have been developed to elucidate it. Such hypotheses include the two-hit hypothesis and the multifactor-hit hypothesis, both of which underline a more-than-one-step process of NAFLD development [73].

Studies have shown that NASH induces cytochrome P450 2E1 (CYP2E1) [72], an enzyme, metabolizing hydrophobic compounds and organic solvents, which include hepatotoxic agents such as CCL4 [74]. Other factors such as ethanol, fasting, diabetes, obesity, and a high-fat/low-carbohydrate diet also induce CYP2E1, which in turn generates reactive oxygen species (ROS) in hepatic mitochondria and ER [75] and induces lipid peroxidation [76]. The subsequent imbalance between oxidants and antioxidants (oxidative stress) leads to liver injury and inflammation [75].

ZE treatment in the NASH animal model reduced lipid peroxidation levels and oxidative stress in a dose-dependent manner [58]. In one population-based research in China, 2,935 participants had their serum carotenoid levels analyzed. The prevalence of NAFLD among the middle-aged and old participants was 50.6%, and NAFLD severity was determined by abdominal ultrasonography. Assessment of serum ZE and its isomer LU (ZE + LU) showed that high serum levels of ZE + LU significantly and inversely correlated with NAFLD severity [77].

In a superimposed nonobese NASH and chronic HBV model, wild-type and hepatitis B virus (HBV) transgenic mice were treated with 2 mg/kg ZD three times a week for eight weeks [60]. This treatment was shown to partially restore the animal body weight and reduced liver-to-body weight ratio. More importantly, ZD reduced steatosis in hepatocytes, inflammation, and fibrosis and restored liver function. Furthermore, ZD upregulated the gene expression of antioxidant enzymes catalase (CAT) and superoxide dismutase 1 (SOD1) and reduced the activity of oxidative stress biomarkers 3-nitrotyrosine (3-NTR) and malondialdehyde (MDA) [60]. Studies [78, 79] have showed a positive correlation between elevated proinflammatory cytokines, such as TNF-*α*, and the occurrence of NAFLD. The levels of chemokine monocyte chemoattractant protein 1 (MCP-1) were also elevated in simple steatosis patients [80]. Treatment with ZD lowered all proinflammatory cytokines and chemokines such as TNF-*α*, IL-1*ß*, IL-6, and MCP-1 and reduced the activities of caspase 3/7 and 8 [60]. These findings highlighted the potential antioxidative, anti-inflammatory, and antiapoptotic roles of ZD in the treatment of NASH.

### 3.3. Hepatitis B Virus

HBV is a hepatotropic noncytopathic DNA virus leading to acute and chronic infections. Globally, 250 million people are estimated to have chronic HBV. Hepatitis B-related complications include cirrhosis and HCC, which are fatal and account for more than half million of death per year [81, 82]. Different genotypes of HBV exist and influence the formation of hepatitis B surface antigen (HBsAg) as well as HBV DNA's intracellular and extracellular levels. These genotypes may also cause different degrees of damage in hepatocytes and determine the disease prognosis [82, 83]. Overall, chronic hepatitis B virus has no cure despite development of novel antiviral and immune therapies [84]. Inducing NASH in the transgenic HBV mice model reactivated HBV replication, while ZD administration reduced HBV DNA replication and serum HBsAg levels [60]. These preliminary results call for further investigation as ZD could be a potential therapeutic agent in the treatment of viral hepatic infections.

### 3.4. Alcoholic Fatty Liver Disease

Like NAFLD, alcoholic fatty liver disease (AFLD) is characterized by steatosis with or without inflammation. The etiology however differs in that AFLD is alcohol-induced. More broadly, alcoholic liver disease (ALD) refers to a range of ethanol-induced liver injuries, such as steatosis, alcoholic hepatitis, and cirrhosis, caused by excessive consumption of alcohol (>20–30 g/day). Alcohol abstinence is prescribed in all stages of ALD. Survival rate and increased risk of developing cirrhosis are seen in patients who fail to abstain from alcohol and manage their lifestyle after diagnosis [85, 86]. Histological stages of ALD often include simple steatosis, alcoholic hepatitis, and chronic hepatitis with fibrosis or cirrhosis [87]. In addition to alcohol consumption, risk factors of developing ALD include genetic predisposition, ethnicity, diet, obesity, concurrent viral infections, and gender [85, 87].

At the hepatocyte levels, ethanol is converted into acetaldehyde with the help of the oxidizing enzymes alcohol dehydrogenase (ADH) and CYP2E1 [88]. The activation of CYP2E1 to oxidize ethanol increases oxidative stress by producing ROS and reactive nitrogen species (RNS). Chronic ethanol-induced reactive species alter the morphology and function of mitochondria and may lead to hepatocyte apoptosis [89]. To counter ethanol-induced hepatotoxicity, damaged mitochondria are eliminated by mitophagy through the PINK1-Parkin pathway [90]. Moreover, evidence suggests that this CYP2E1-meditated oxidative stress reduces the phosphorylation of Akt in the liver, which in turn induces steatosis [91]. In this process, intestinal permeability to endotoxin increases [92], and liposaccharides (LPS) from Gram-negative bacteria leak into the blood circulation and trigger the release of macrophages in the blood and liver. These macrophages secrete cytokines such as TNF*α*, IL-1*β*, and IL-6 and trigger NF-*κ*B [93]. The activation of Kupffer cells in the liver eventually lead to liver fibrosis.

As previously mentioned, ZD attenuated liver fibrosis and improved hepatic histology in the AFLD animal model [59], where it was reported to modulate MAPK pathways (i.e., p38 MAPK, ERK, and JNK) to reduce ethanol-induced hepatotoxicity. Here, ZD inhibited the phosphorylation of p38 MAPK and JNK, which had been increased with chronic ethanol administration. On the contrary, the inhibition of p38 MAPK and ERK (but not JNK) impaired the therapeutic effect of ZD. Furthermore, the carotenoid (25 mg/kg) reinstated the animals' body weight, restored the expression levels of SOD1 and CAT, as in the NASH animal model [60], and lowered the expression of cytochrome CYP2E1. Inhibition of CYP2E1 was shown to decrease highly reactive free radicals and protect against liver injury in other studies [94–96], possibly through the HMGB1-TLR4 signaling pathway [97, 98]. Similarly, ZD administration reduced the oxidative stress products serum MDA and plasma 8-isoprostane. ZD also inhibited the activity of NF-kB, by restoring NF-kB cytosolic inhibitor-kappa B-alpha (IkBa), and downregulated the expression levels of TNF*α*, IL-1*β*, IL-6, and MCP-1. Finally, as ethanol induces hepatocyte apoptosis, ZD downregulated the mRNA expressions of proapoptosis genes Bax-1 and caspase-3/7 and upregulated the expression of Bcl-2 [59]. Another study [99] established the ability of ZD to ameliorate liver histology in the ALFD model and decrease serum levels of ALT and AST. The authors showed ZD binding to two receptors, P2X7 and adipoR1, which in turn induced mitophagy. The role of ZD-modulating autophagy was then verified by inhibiting systemic autophagy and specific receptors P2X7 and adipoR1, which impaired the therapeutic effect of ZD. After binding to P2X7 and adipoR1, ZD inhibited PI3K/AkT and activated the AMPK/FoxO3a pathway, which restored mitophagy and inactivated the NLRP3 inflammasome pathway. Inactivation of NLRP3 decreased caspase-1 activity as well as IL-1*ß* and TN-Fa and restored cellular autophagy through the upregulation of the gene expressions of Atg5, beclin-1, and LC3A/B and the downregulation of p62, caspase-3/7, and caspase-8. Overall, as in the NASH model, ZD played antioxidant, anti-inflammatory, antihepatotoxic, and antiapoptotic roles in the treatment of AFLD.

### 3.5. Hepatocellular Carcinoma

Hepatocellular carcinoma (HCC) is the most common type of primary liver cancer and the third leading cause of cancer mortality worldwide with about 6-month life expectancy from the time of diagnosis. HCC is associated with chronic liver diseases with HBV infection being the most common risk factor. Other risk factors include HCV, cirrhosis, and diabetes. Geographically, HCC incidence has been higher in regions with widespread of HBV and HCV, such as East Asia, combined with chronic alcoholism or consumption of aflatoxin B1-contaminated peanuts. In the US, HCC incidence and mortality have been increasing across different demographic groups and were associated with a medical history of HBV and HCV [100, 101]. Treatment options for early-stage HCC involve liver resection, transplantation, percutaneous ablation, and chemoembolization; however, early diagnosis remains difficult [102]. The pathogenesis of HCC is a multistep process involving alteration of gene expression. Moreover, HBV and HCV, with chronic aflatoxin exposure or excessive alcohol consumption, produce inflammatory responses, which generate ROS [101].

*In vitro*, rat ascites hepatoma AH109 A cells were cocultured with rat mesothelial cells. The addition of ZE to the culture inhibited AH109 invasion. This inhibitory effect increased when AH109 cells were pretreated with ROS-generating enzymes before adding ZE. In addition, pretreating AH109 with ZE before culturing AH109 with mesothelial cells lowered AH109 invasive activities. The authors of the study concluded that ZE may remain in the plasma membrane and inhibit HCC invasion through its antioxidant properties [103]. In line with these findings, Woodall et al. [104] showed that ZE protected phospholipids in aqueous solution and liposome against lipid peroxidation, which could be through allylic hydrogen abstraction. This involves the transfer of a hydrogen atom from ZE to the radical for radical stabilization [105]. Moreover, compared with *β*-carotene, astaxanthin, and lycopene, zeaxanthin offers the greatest protection against lipid peroxidation in liposomes [104]. These results support the antioxidant properties of ZE. So far, and to our best knowledge, there are no published studies on the effect of ZD on HCC.

### 3.6. Liver Failure

Liver failure (LF) is a fatal condition in which the liver loses its functions. The disease can be categorized into acute liver failure, acute-on-chronic liver failure, and decompensated liver cirrhosis. Acute LF is a severe impairment of liver function marked by hepatic encephalopathy and bleeding as result of liver necrosis. This condition has an acute onset (within 6 months) and can be caused by viral infections, autoimmune hepatitis, and drug allergy-induced liver damage [106]. Acute-on-chronic LF is an acute liver deterioration of pre-existing chronic liver disease. Bernal et al. [107] divides it further into three types: type A includes noncirrhotic condition which histologically differs from acute LF by the presence of substantial hepatic fibrosis, type B compensated cirrhosis with hepatic deterioration after a major insult, and type C occurs in patients with previous or concurrent cirrhotic decompensation. Finally, cirrhosis occurs following chronic liver injury, which produces extensive fibrosis. Most cirrhotic patients are asymptotic until they develop decompensated cirrhosis, in which case patients may have symptoms such as ascites, hepatic encephalopathy, and variceal bleeding [108].

All liver failure cases are accompanied by poor prognosis, and this often calls for liver transplantation as the only life-saving intervention. Liver transplantation faces many challenges such as organ availability and timing of surgery. One potential treatment for acute LF is stem cell therapy, in which differentiated hepatocyte-like cells are transplanted in the failing liver where stem cells can recover essential hepatic functions [109]. However, this therapy have so far resulted in low cell survival after transplantation due to the stem cells' inability to adapt to an impaired liver environment, in which they fail to resist oxidative and inflammatory stress [110].

Treating human adipose-derived mesenchymal stem cells (hADMSC) with ZD before transplantation increased stem cell survival and improved the therapeutic outcome [111]. Incubation of stem cells with ZD (0.5 *μ*m), in a differentiation medium, inhibited inflammation and reactive oxygen species (ROS) production via the PKC/Raf-1/MAPK/NF-kB pathway, which upregulated microRNA-210, and maintained cell homeostasis. Here, ZD reduced TNF-*α* and IL-6 as well as the activity of caspases 3/7 and 8 and recovered the GSH/GSSG ratio and CAT/SOD1 protein expression. ZD also increased the secretion of IL-10, a hepatocyte growth factor (HGF), and indoleamine 2,3-dioxygenase (IDO) from stem cells and promoted their differentiation into hepatocytes. This was shown by the increased levels of proteins *α*-fetoprotein (AFP) and hepatocyte nuclear factor 4*α* (HNF4), the improved expression of liver-regeneration genes, epidermal growth factor (EGF) and oncostatin M (OSM), and the enhanced secretion of urea and albumin. Finally, the study highlighted the potential role of ZD in optimizing stem cell therapy [111].

### 3.7. ZD Safety

All reviewed papers reported no side effects from ZD and ZE treatment. Toxicological studies involving ZE support these findings [112].

## 4. Future Challenges and Prospects

**Figure 3:**
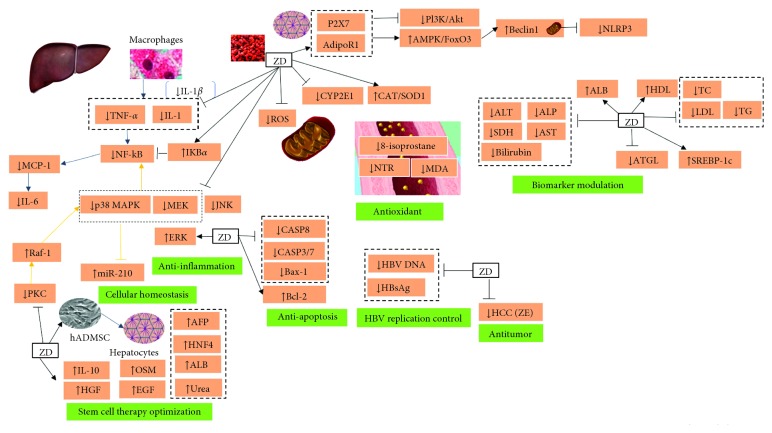
Molecular mechanisms of action of zeaxanthin dipalmitate.

The literature on ZD and its nonesterified form ZE in the treatment of liver disease is limited regardless of the safety and potential therapeutic effects of this carotenoid (Table 2, Figure 3). Nonetheless, as the trend of investigating the properties of herbal active compounds is on the rise and with goji berry's increasing popularity and commercialization as a superfood, ZD will likely get more attention from the scientific community, and its effect on liver disease might be studied further.

## 5. Conclusion

The carotenoid ZD yields very promising applications in the treatment of liver disease. Although research has shown its safety and antioxidant, hepatoprotective, antiapoptotic, antifibrotic, antitumor, and anti-inflammatory qualities, further studies are needed, especially clinical ones to investigate the efficacy of ZD in treating patients with liver disease.

## Figures and Tables

**Table 1:** Quantification methods of zeaxanthin dipalmitate in goji berry.

	Li et al. [39]	Weller et al. [40]	Peng et al. [41]	Chang et al. [37]	Karioti et al. [38]	Zheng et al. [36]	Patsilinakos et al. [42]
Study aim	Compare different eluents for optimal carotenoid extraction	Evaluate ZE esters, free ZE, and total ZE content in plant extracts as potential source of oleoresins	Quantify ZD in different goji berry species	Extract ZD	Achieve exhaustive extraction of total carotenoid and ZD	Compare different eluents for optional extraction of total carotenoid content	Use simplified extraction method of carotenoids/conduct colorimetric analysis
Goji berry samples	Fruit, freeze-dried	Fruit, heat-dried	Fruit, 2 g in 5 ml of water for 30 min and then ground	Fruit, freeze-dried/ground	Fruit, N_2_-freeze-dried and ground; juice; jam	Goji berry juice	Fruit, frozen and then unfrozen before use
Extraction	Petroleum ether/acetone (1 : 1)	Methanol/ethyl acetate/light petroleum (1 : 1 : 1)	Sonication petroleum ether/acetone extraction	Ultrasonic acetone extraction	Dried fruit: ultrasonication hexane/acetone (50 : 50)	Hexane/ethanol/acetone (2 : 1 : 1)	Ethanol-acidified water mixture/cyclohexane (1 : 1); ethyl acetate
					Juice: ultrasonication hexane/acetone (40 : 60)		
HPLC solvent	Acetonitrile/methylene chloride (60 : 42)	Petroleum/acetone (94 : 6)	Acetonitrile/dichloromethane (42 : 58)	Dichloromethane	Acetone/methanol (55 : 45)	NA	Acetone/methanol (80 : 20)
Quantification method	HPLC-UV-visible spectrophotometry	HPLC-DAD and LC-(APCI)MS	HPLC-DAD	HPLC + nuclear magnetic resonance spectroscopy	HPLC-DAD	UV-visible spectrophotometry	HPLC-DAD
Instrument	NR	HP 1100 modular system (Agilent 1100)	Agilent 1100	NR	Agilent 1200	Ultrasonic stirring apparatus (25 kHz, 400 W)^*∗*^	PerkinElmer Series 200
Column chromatography	Skim-pack CLC-ODS (150 mm × 6 mm i.d.) with C18 reversed-phase column	YMC (5 *μ*m, 250 × 4.6 mm i.d.) with C30 reversed-phase (10 × 4.0 mm i.d.) precolumn	Alltima C18 (5 *μ*m, 250 mm × 4.6 mm i.d.)	(30 cm × 3 cm i.d.) and (30 cm × 10 cm i.d.)	Luna RP-C18 (5 *μ*m, 150 mm × 4.6 mm)	NA	Luna (Phenomenax) PR-C18 (5 *μ*m, 250 mm × 4.6 mm)
ZD recrystallization	NA	NA	NA	One sample used liquid antisolvent ethanol; another used CO_2_ supercritical antisolvent precipitation	NA	NA	NA
Saponification (Y/N)	N	Y, in sample intended to measure total zeaxanthin. Methanolic KOH used	N	N	N	Y, compared ZD content in saponified and nonsaponified samples	N

^*∗*^As reported by the authors. NR: not reported; NA: not applicable; Y/N: yes/no.

**Table 2:** Studies using zeaxanthin dipalmitate and zeaxanthin.

Experimental drug	Model	Experiement duration	Dose (ZD or ZE) and course of treatment	Case/control	Disease type	Method	Efficacy	Control	ZD side effect	Reference	Reference #
ZD	Hepatocytes of Wistar rats	25.5 hours	0.1 *μ*M; 1 *μ*M; 10 *μ*M	NA	ALD/ethanol-induced liver injury	Cell culture in 10 nM CCI4-containing medium	Inhibited ALT and SDH in a dose-dependent manner	Unchallenged and untreated cells	None	Kim	66
ZE			0.1 *μ*M; 1 *μ*M; 10 *μ*M	NA	ALD/ethanol-induced liver injury	Cell culture in 10 nM CCI4-containing medium	Inhibited ALT and SDH in a dose-dependent manner	Unchallenged and untreated cells	None		
ZD	Wistar rats	6 weeks	12.5 mg/kg/d; 25.0 mg/kg/d	16/5	Hepatic fibrosis (secondary biliary fibrosis	Bile duct ligation	25 mg/kg significantly reduced collagen content in the liver. TBARS decreased. Elevated serum ALT significantly decreased at 25.0 mg/kg	Sham operation	None	Kim	57
ZE	Mongolian gerbils	6 weeks	12.5 mg/kg/d; 25.0 mg/kg/6	18/6	NASH	MC diet	Decreased oxidative stress and liver fibrosis in a dose-dependent manner. 25 mg/kg/d inhibited fibrosis	Regular chow	None	Chamberlain	58
ZE + LU	Population-based study	4 year and 8 months: July 2008–June 2010 and April 2011–January 2013	NA	2,935/NA	NASH	NA/Regular diet	High serum levels of ZE + LU significantly and inversely correlated with NAFLD severity	NA	None	Cao	77
ZD	Transgenic balb/c mice; wild-type balb/c mice	8 weeks	2 mg/kg for three days a week	70/10	NASH + HBV	MC diet and HBV transgenic mouse phenotype	Restored body weight. Inhibited oxidative stress. Regulated liver enzyme levels. Lowered production HBV DNA replication and serum HBsAg levels	Regular chow	None	Li	60
ZD	Sprague–Dawley rats	10 weeks	25 mg/kg/d from 5 to 10th week	18/6	AFLD	Intragastrically fed 4.0 g/kg ethanol diluted in water for 10 weeks	Restored body weight. Improved liver histology. Decreased inflammation and apoptosis. Reduced hepatic oxidative stress. Did not change blood alcohol level	Regular chow	None	Xiao	59
ZD	Sprague–Dawley rats; BRL-2A cells	6 weeks	Experiments: *in vitro*: 1 *μ*M. AFLD animal model: 10 mg/kg/d for 2 weeks; systemic autophagy inhibition model: 10 mg/kg/d for 6 weeks; knockdown of P2X7 and AdipoR1: 10 mg/kg/d for 6 weeks	48/16 (not clear)	AFLD	Lieber-DeCarli liquid diet with ethanol intake increased gradually and maintained at 5% (w/v)	Induced mitophagy. Inhibited the P13 K/AKT pathway and restored AMPK/Fox3a. Partially inhibited NLRP3 inflammasome	Dextrin/maltose-based liquid diet	None	Gao	99
ZE	AH109A cells	2 months	5 *μ*M	NA	Hepatocellular carcinoma	AH109 A cells cultured in Donryu rats' peritoneal cavity and harvested for in vitro assays	Inhibited AH109 A cell invasion through its antioxidant properties. ZE may remain in the cell membrane	Control medium 0.5% DSMO alone	None	Kozuki	103
ZD	Human adipose-derived mesenchymal stem cells (hADMSC); nonobese/diabetic severe combined immunodeficient (NOD/SCID) mice	1 week	0.5 *μ*M	84/12	Liver failure	Intraperitoneal injection of nonobese diabetic severe combined immunodeficient mice with pretreated stem cells	Optimized stem cell therapy in animal model. Improved stem cell visibility ratio and decreased apoptosis. Upregulated miR-210 expression. Reduced the activity of caspases 3/7 and 8 and recovered GSH/GSSG and CAT/SOD1 levels	Intraperitoneal injection with PBS only	None	Liu	111

## Supplementary Materials

Supplementary Materials Graphical abstract.
