# Supplementary material for: Zeaxanthin Dipalmitate in the Treatment of Liver Disease
Source: Evid Based Complement Alternat Med. 2019 Aug 21;2019:1475163. doi: 10.1155/2019/1475163 (PMC6721266; doi:10.1155/2019/1475163)
Supplement: Supplementary Materials — Graphical abstract. [file 1475163.f1.pptx]

## Slide 1
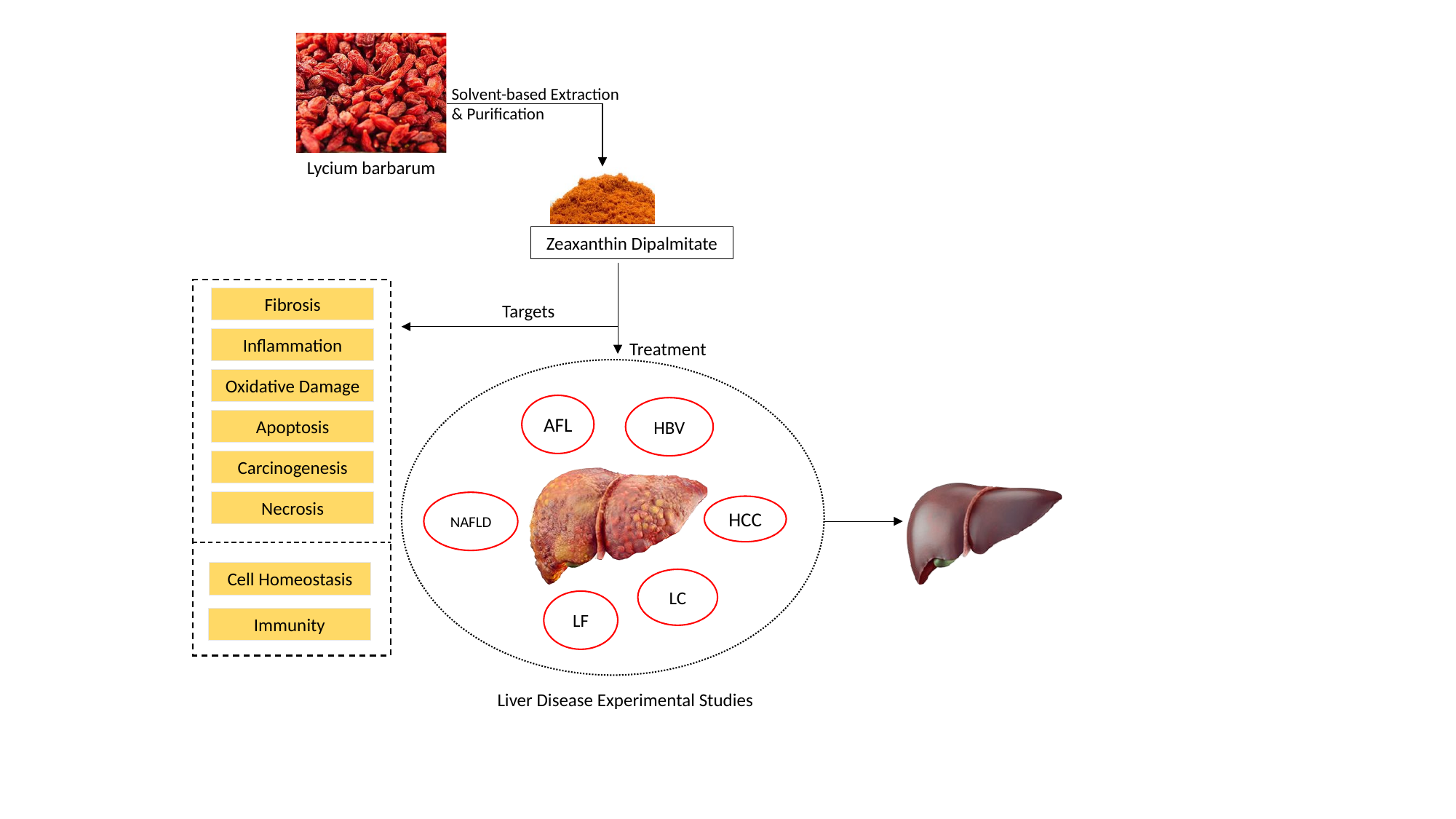

Solvent-based Extraction
& Purification
Lycium barbarum
Zeaxanthin Dipalmitate
Fibrosis
Targets
Inflammation
Treatment
Oxidative Damage
AFL
HBV
Apoptosis
Carcinogenesis
Necrosis
NAFLD
HCC
Cell Homeostasis
LC
LF
Immunity
Liver Disease Experimental Studies
